# Complexation of a guanidinium-modified calixarene with diverse dyes and investigation of the corresponding photophysical response

**DOI:** 10.3762/bjoc.15.139

**Published:** 2019-06-25

**Authors:** Yu-Ying Wang, Yong Kong, Zhe Zheng, Wen-Chao Geng, Zi-Yi Zhao, Hongwei Sun, Dong-Sheng Guo

**Affiliations:** 1 College of Chemistry, Key Laboratory of Functional Polymer Materials, State Key Laboratory of Elemento-Organic Chemistry, Tianjin Key Laboratory of Biosensing and Molecular Recognition, Nankai University, Tianjin 300071, P. R. China; 2 Research Institute of Petroleum Engineering, Sinopec, Beijing 100101, P. R. China

**Keywords:** calixarene, host–guest complexation, luminescent dyes, macrocycles, photophysical properties

## Abstract

We herein describe the comprehensive investigation of the complexation behavior of a guanidinium-modified calix[5]arene pentaisohexyl ether (GC5A) with a variety of typical luminescent dyes. Fluorescein, eosin Y, rose bengal, tetraphenylporphine sulfonate and sulfonated aluminum phthalocyanine were employed as classical aggregation-induced quenching dyes. 2-(*p*-Toluidinyl)naphthalene-6-sulfonic acid and 1-anilinonaphthalene-8-sulfonic acid were selected as representatives of intramolecular charge-transfer dyes. Phosphated tetraphenylethylene was involved as the classical aggregation-induced emission dye. Sulfonated acedan representing one example of two-photon fluorescent probes, was also investigated. A ruthenium(II) complex with carboxylated bipyridyl ligands was included as a representative candidate of luminescent transition-metal complexes. We determined the association constants of the GC5A–dye complexes by fluorescence titration and discuss the complexation-induced photophysical changes. In addition, a comparison of the complexation behavior of GC5A with that of other macrocycles and potential applications according to the diverse photophysical responses are provided.

## Introduction

**Scheme 1:**
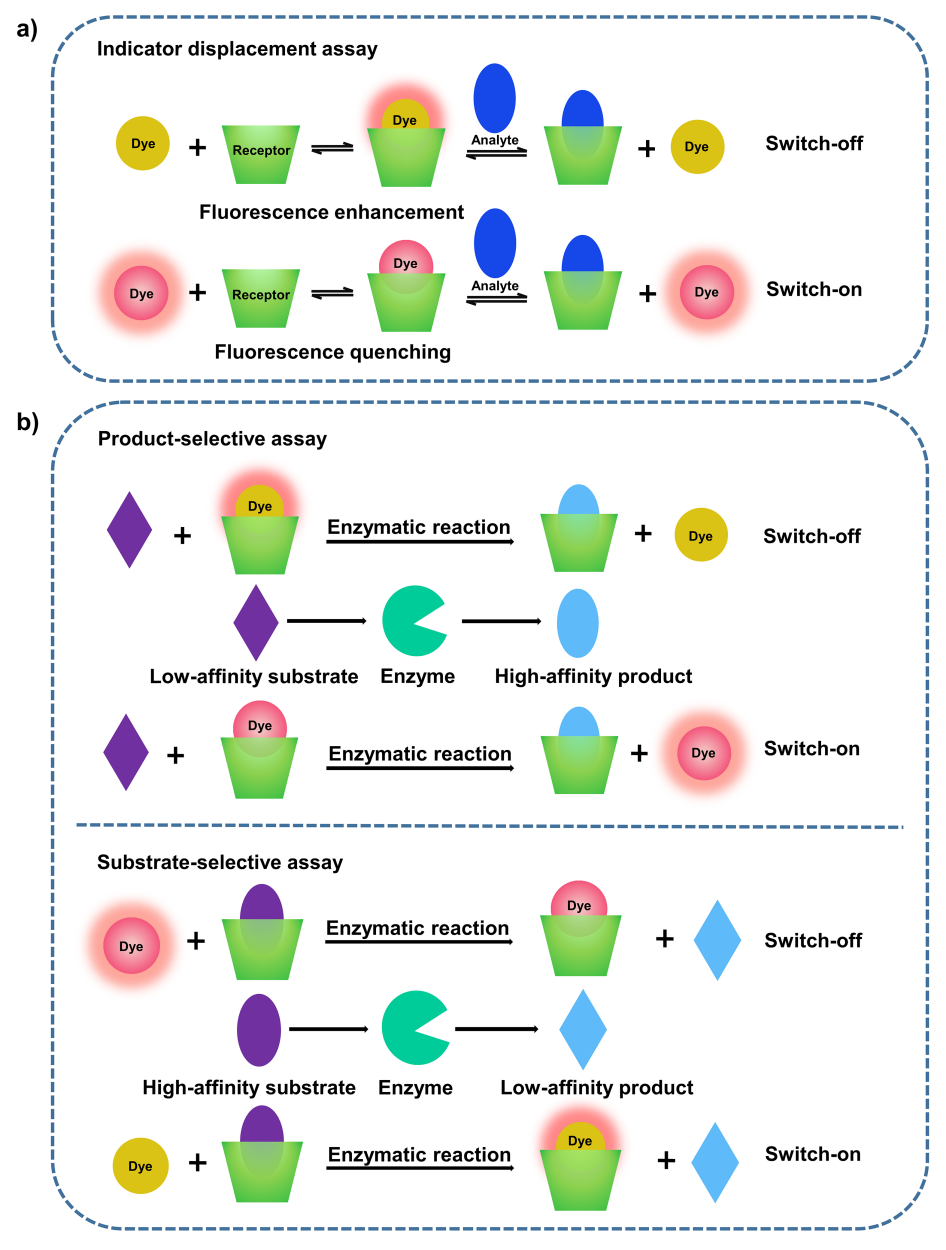
(a) Schematic illustration of IDA. The addition of an analyte competitor leads to switch-on or switch-off sensing, depending on the complexation-induced photophysical alternation of the dye by the receptor. (b) Schematic illustration of STA, divided into two types. A product-selective assay (top) is established when the product (blue) has a higher affinity to the receptor than the substrate (purple); conversely, a substrate-selective assay (bottom) is established when the receptor binds more strongly to the substrate (purple) than to the product (blue).

Fluorescence sensing represents a powerful detection methodology due to its low cost, ease of use and high sensitivity, and has been widely used in fields of chemistry, biomedicine, environment, and so on [1]. Generally, the conversion of a luminescent dye to a chemosensor requires a grafting recognition motif for a particular analyte, whose installation often involves laborious and time-consuming syntheses. Alternatively, supramolecular chemistry provides a non-covalent approach to achieve fluorescence sensing [2]. An elegant supramolecular strategy, named indicator displacement assay (IDA), was established and popularized by Anslyn and co-workers (Scheme 1a) [3–4]. The complexation of a luminescent dye by a suitable receptor leads to a readily detectable change of its luminescence property, prominently its intensity. An analyte then displaces the luminescent dye from the complex, resulting in a detectable luminescence response converting a receptor–analyte binding event into an easily observable signal. Subsequent to IDA, Nau and co-workers conceptualized a novel approach towards enzyme assays, termed supramolecular tandem assay (STA) (Scheme 1b) [5]. STA is envisaged as a time-resolved version of IDA and the key idea is that the competitor is not added, but rather created during the course of an enzymatic reaction. Thus, the progress of the reaction can be signaled by a luminescence increase or decrease with time, which enables a highly sensitive real-time luminescence monitoring of the enzymatic activity [6]. STA has been applied to screening enzyme inhibitors or activators, determining absolute concentrations of analytes, and developing a sensor array [7–8]. The key factor in these assay approaches is the development of reporter pairs. An ideal reporter pair should fulfill two issues: a) the designed receptor binds the analyte strongly and selectively and b) the competitive binding of the analyte gives rise to an extraordinary luminescence response of the reporter dye.

Macrocyclic hosts constitute a family of well-studied artificial receptors with a discrete cavity that is selective for complementary binding to certain guests [9]. Modulation of properties of organic fluorophores through supramolecular encapsulation using artificial macrocyclic hosts has always been an active research area [10–12]. The complexes of macrocycles with luminescent dyes not only act as reporter pairs for sensing [13–15], but also offer various applications in bioimaging [16–17], constructing supramolecular dye lasers [18–19], protecting fluorophores from photodegradation [20–21] and manufacturing organic luminescent materials [22–24]. Also, variations of luminescence have been utilized to obtain information about the hydrophobicity, polarity, and polarizability of the inner supramolecular cavities [25].

Calixarenes are the third generation of macrocyclic compounds composed of phenolic units bridged with methylene groups at the *o*-positions of phenolic hydroxy groups [9]. We have focused on molecular recognition and self-assembly of water-soluble calixarene derivatives for a long time, directed by exploring biomedical applications of these compounds. Recently, we developed a series of guanidinium-modified calixarenes as novel artificial receptors [13–14]. We achieved ultrasensitive and specific fluorescence detection of lysophosphatidic acid (a cancer biomarker) by executing fluorescent IDA with guanidinium-modified calix[5]arene pentaisohexyl ether (GC5A) and fluorescein (Fl) as the reporter pair [26]. The ultrasensitive detection is feasible for diagnosing ovarian and other gynecologic cancers at their early stages. Also, we proposed a nanoplatform where the fluorescence and photoactivity of photosensitizers (PSs) were annihilated by the complexation of guanidinium-modified calix[5]arene pentadodecyl ether while reactivated by adenosine triphosphate (a cancer biomarker) displacement. This novel supramolecular phototheranostics strategy realized both tumor-selective imaging and targeted therapy in vivo [27]. These biomedical applications depend on the fine set-up of receptor–dye complexation. In this work, we comprehensively investigated the complexation behavior of GC5A with a series of luminescent dyes. Their complexation-induced photophysical alternations were comparatively discussed. The established toolbox of reporter pairs is expected to shed light on applications in the areas of IDA and STA methodologies, and the obtained fundamental insights are prerequisite for future applications in imaging, lasing, activatable phototheranostics, manufacturing organic luminescent materials and constructing dye-sensitized solar cells.

## Results and Discussion

**Scheme 2:**
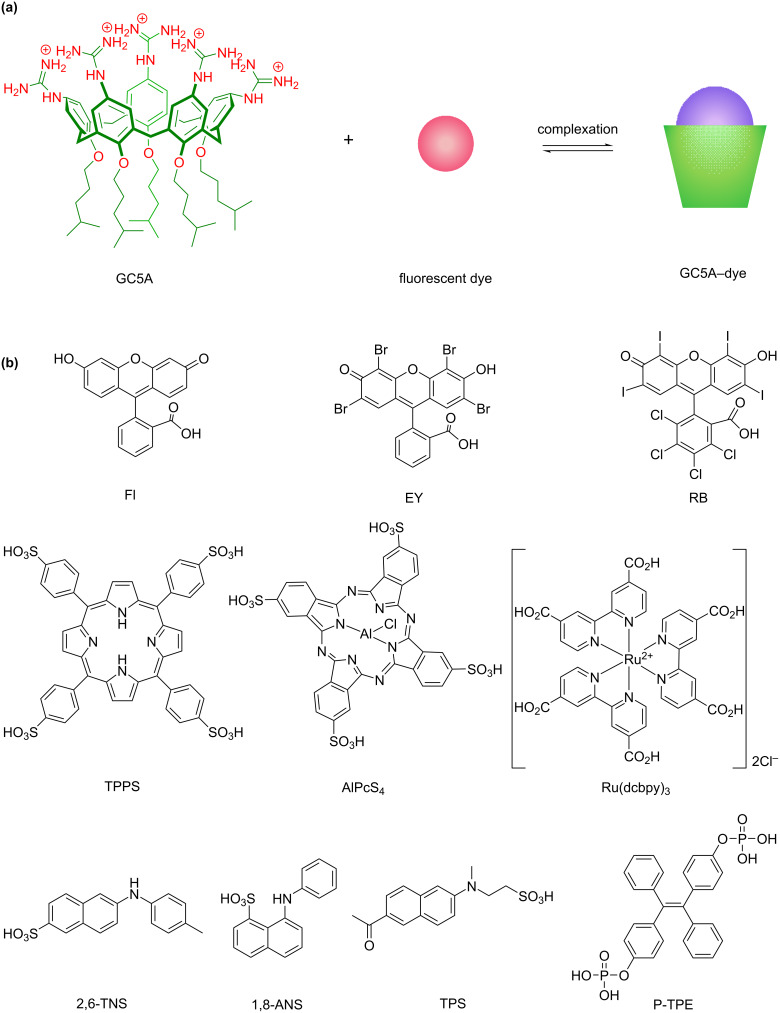
(a) The chemical structure of GC5A and schematic illustration of the binding between the luminescent dye and GC5A. (b) Chemical structures of luminescent dyes employed in this work.

GC5A was prepared according to our previous procedure and represents a robust water-soluble macrocyclic receptor [26]. According to the positively charged feature of GC5A, a series of negatively charged dyes, including Fl, eosin Y (EY), rose bengal (RB), tetraphenylporphine sulfonate (TPPS), sulfonated aluminum phthalocyanine (AlPcS_4_), 2-(*p*-toluidinyl)naphthalene-6-sulfonic acid (2,6-TNS), 1-anilinonaphthalene-8-sulfonic acid (1,8-ANS), phosphated tetraphenylethylene (P-TPE), sulfonated acedan (TPS), and a ruthenium(II) complex with carboxylated bipyridyl ligands (Ru(dcbpy)_3_), were screened as model guests on account of the desired strong host–guest binding affinity (Scheme 2). Fl, EY, RB, TPPS and AlPcS_4_ were employed as classical aggregation-caused quenching (ACQ) dyes; 2,6-TNS and 1,8-ANS were selected as intramolecular charge-transfer dyes. P-TPE was included in the study as a classical aggregation-induced emission (AIE) dye and TPS as a representative of a two-photon fluorescent probe. Ru(dcbpy)_3_ was involved as a member of luminescent transition-metal complexes. Of our special interest in the present study is to disclose photophysical changes of various dyes upon complexation by GC5A, to understand the physicochemical property of the GC5A cavity through the comprehensive discussion, and further guide to potential applications of these complexes.

### The complexation of GC5A with ACQ dyes

Conventional fluorophores often emit strongly in their dilute solution as isolated molecules, but once aggregation occurred, fluorescence is quenched owing to intermolecular packing. These ACQ dyes generally possess large conjugated coplanar molecular structures which lead to strong intermolecular interactions. Fl is one of the most common high-performance fluorescent reagents routinely used in biological research for labeling and detection. It is characterized by high absorptivity, excellent brightness, a relatively good water solubility and possesses ACQ properties. In our previous work, we have reported the complexation behavior between GC5A and Fl [26]. The formation of a ground-state complex was confirmed by UV–vis titration, which revealed significant changes in band shapes and intensities. Upon fluorescence titration, the intrinsic emission of Fl was drastically quenched by gradual addition of GC5A. Given calixarenes as well-demonstrated fluorescence quenchers acting through a photoinduced electron transfer (PET) mechanism [8,28–29], we hypothesize an electron transfer-induced quenching in the GC5A–Fl complex as underlying mechanism. The binding stoichiometry between GC5A and Fl was determined to be 1:1 according to the Job's plot. The association constant (*K*_a_) was well fitted as 5.0 × 10^6^ M^−1^ (Table 1) by a 1:1 binding model according to both fluorescence titration and UV–vis titration. The drastically altered fluorescence signal upon complexation, *I*_free_/*I*_bound_, is calculated as a factor of 37.

EY, RB, TPPS and AlPcS_4_ are four commonly used PSs in photodynamic therapy with ACQ features. Upon light excitation, PSs are excited from the singlet ground state to the singlet excited state and then to a triplet excited state via intersystem crossing. PSs at their excited triplet states are able to react with substrates, typically oxygen, to produce reactive oxygen species. The competitive complexation can not only be applied in diagnosis via the aforementioned IDA but also be engaged in therapy. We proposed a host–guest strategy for activatable phototheranostics termed biomarker displacement activation (BDA) [27]. In that work, we employed the four PSs and studied their host–guest complexation with GC5A. Each PS has a strong binding affinity upon 1:1 complexation with GC5A (Table 1), accompanied by a drastic annihilation of both fluorescence and photoactivity (reactive oxygen species generation). We studied the photophysical response upon complexation in detail taking AlPcS_4_ as an example. The emission peak of AlPcS_4_ remains unshifted when the overall fluorescence intensity is reduced by addition of GC5A, indicating that the singlet excited state of AlPcS_4_ returns to the ground state through a PET pathway. The fluorescence lifetime of AlPcS_4_ remains unaltered after addition of GC5A, indicating the absence of dynamic quenching of the residual uncomplexed AlPcS_4_ and therefore confirming the formation of a statically quenched complex. UV–vis titration further verified the formation of a ground-state complex, with drastic changes in both, band shapes and intensities. The calculated rate constant of PET is faster than that of fluorescence and intersystem crossing, which makes it a more favorable deactivation pathway of the first excited singlet state leading to both fluorescence quenching and photoactivity annihilation.

**Table 1:** Binding constants and binding stoichiometries of GC5A–dye complexes.

Dye	*K*_a_ [M^−1^]	Binding stoichiometry^a^	Ref.

Fl	5.0 × 10^6^	1:1	[26]
EY	5.7 × 10^8^	1:1	[27]
RB	9.6 × 10^7^	1:1	[27]
TPPS	1.1 × 10^8^	1:1	[27]
AlPcS_4_	1.7 × 10^8^	1:1	[27]
1,8-ANS	(3.0 ± 0.6) × 10^6^	1:1	this work
2,6-TNS	(4.4 ± 1.6) × 10^6^	1:1	this work
P-TPE	(8.4 ± 1.1) × 10^7^	1:1	this work
TPS	(1.4 ± 0.1) × 10^6^	1:1	this work
Ru(dcbpy)_3_	(9.1 ± 0.4) × 10^7^	3:1	this work

^a^Binding stoichiometry of host to guest.

The complexation behavior between some of abovementioned luminescent dyes and cyclodextrin (CD) derivatives, especially β-CD, also has been investigated in the literature [30–32]. β-CD exhibits relatively weak binding affinities on the magnitude (or less than) of 10^3^ M^−1^ to Fl, RB and TPPS, accompanied by a slight fluorescence enhancement [10,33–34]. One extraordinary example is the complexation between permethylated β-CD (PMCD) and TPPS. A specific 2:1 PMCD–TPPS complex was formed with an exceptionally high binding constant which was even too large to be calculated in aqueous solution [35]. The fluorescence intensity of TPPS was slightly enhanced upon PMCD encapsulation, accompanied by a sharpening of the fluorescence band [36]. The 2:1 PMCD–TPPS complex has been applied in photodynamic therapy with improved photoactivity due to the inhibition of TPPS aggregation and the shielding effect of PMCD against quenching of the TPPS triplet state [37]. Also, it has been employed in the construction of photovoltaic materials and light-harvesting systems [38–39]. The complexation of GC5A with ACQ dyes generally has the advantage of strong binding affinities accompanied by a drastic quenching of fluorescence, which is a proper feature to be applied in IDA, product-selective STA and BDA, resulting in a desired switch-on sensing (Scheme 1) or activatable phototheranostics. With respect to the sensing application, the reporter pairs can be used in very dilute solutions benefiting from the high affinities and the corresponding drastic fluorescence quenching, which is desirable from the viewpoints of economy, sensitivity and interference [8,26]. With regard to the treating application, high affinities with PSs avoid undesired off-target leaking during its systemic delivery; “super”-quenching minimizes the imaging background and phototoxicity to normal tissues [27].

### The complexation of GC5A with ICT dyes

1,8-ANS and 2,6-TNS are considered involving ICT processes in their photophysics [40–41] because of their intramolecular electron-donating and electron-accepting structures. An increase in charge separation within ICT probes would occur upon excitation which results in a larger dipole moment in the excited state. The energy of the excited state with a larger dipole moment could be reduced by interaction with a high polarity environment and could be elevated by interaction with non-polar environment. Thus, the S_1_–S_0_ energy gap increases as the polarity decreases resulting in a blue-shifted emission in a hydrophobic environment. Their pronounced solvatochromic effect makes them perfect environmental sensitive probes.

**Figure 1:**
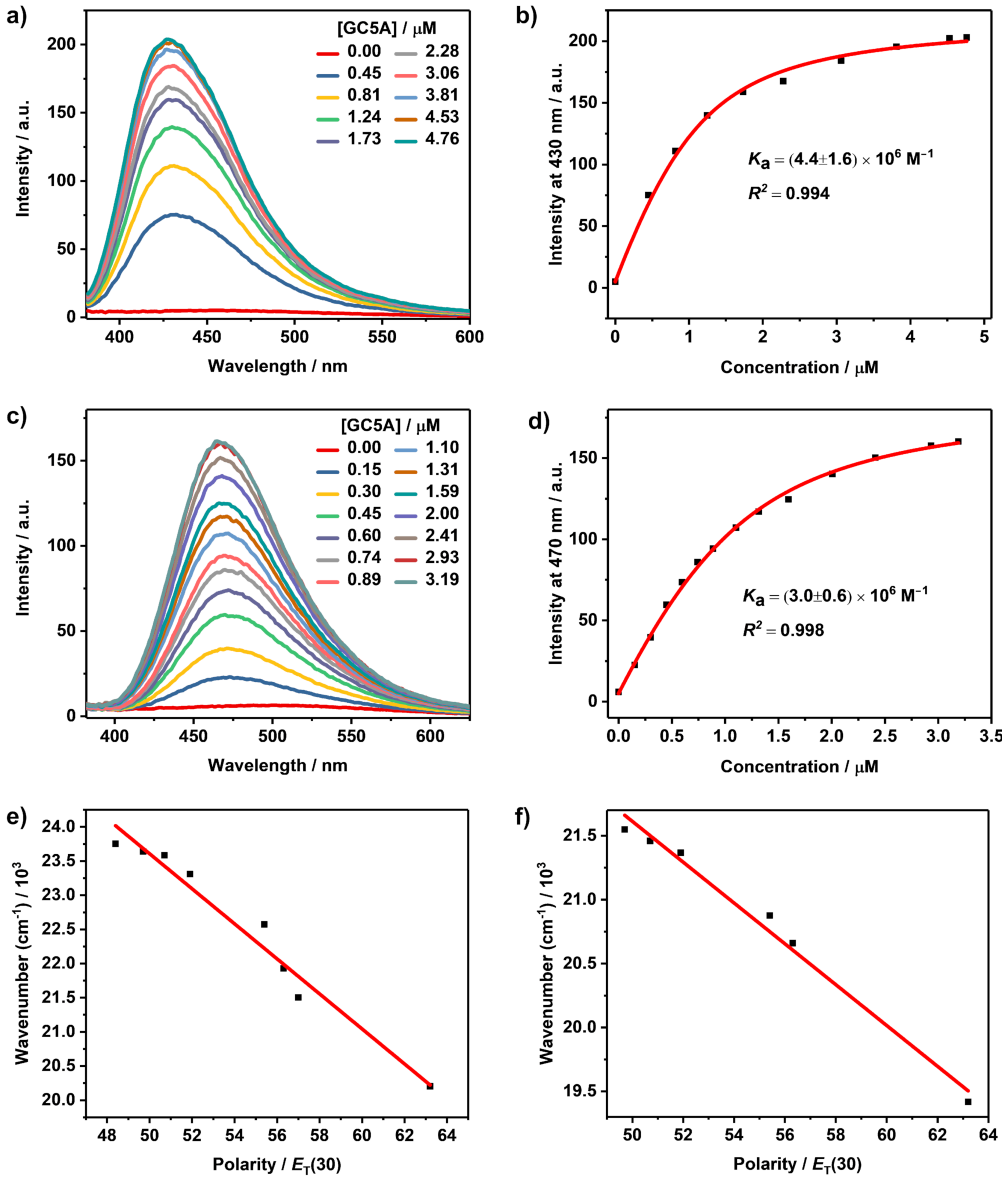
Direct fluorescence titrations (λ_ex_ = 350 nm) of 2,6-TNS (1.0 μM) (a) and 1,8-ANS (1.0 μM) (c) with GC5A in HEPES buffer (10 mM, pH 7.4) at 25 °C and the associated titration curves of 2,6-TNS (λ_em_ = 430 nm) (b) and 1,8-ANS (λ_em_ = 470 nm) (d) fitting according to a 1:1 binding stoichiometry. Emission wavenumbers of 2,6-TNS (e) and 1,8-ANS (f) in different solvents as a function of the *E*_T_(30) polarity scale.

1,8-ANS and 2,6-TNS are essentially non-fluorescent in aqueous solution, but become highly fluorescent in non-polar solvents or when they are bound to proteins and membranes [42–43]. This enhancement is commonly understood in terms of the relocation of the guest into the more hydrophobic environment. The decreased rate constant of internal conversion from S_1_ to S_0_ is responsible for the increase in the fluorescence quantum yield in these hydrophobic environments [44–45]. The fluorescence intensity of 2,6-TNS was extremely low with a maximum emission wavelength at 495 nm in aqueous solution [46]. Its fluorescence was dramatically enhanced upon titration with GC5A (*I*_bound_/*I*_free_ = 39). An emission maximum was observed at 430 nm as shown in Figure 1a. *K*_a_ was fitted as (4.4 ± 1.6) × 10^6^ M^−1^ according to the sequential changes of fluorescence intensity of 2,6-TNS at various concentrations of GC5A (Figure 1b, Table 1). The complexation behavior between 1,8-ANS and GC5A is similar to that of 2,6-TNS. The emission maximum of free 1,8-ANS lies at 515 nm in water [42] and upon complexation with GC5A, its fluorescence was enhanced (*I*_bound_/*I*_free_ = 25) with an emission maximum at 470 nm (Figure 1c). *K*_a_ was fitted as (3.0 ± 0.6) × 10^6^ M^−1^ (Figure 1d, Table 1).

2,6-TNS and 1,8-ANS have been employed to estimate the inner cavity polarity of macrocyclic hosts before [10]. Micropolarity of GC5A was quantified utilizing the polarity empirical index, *E*_T_(30), which is defined as the transition energy for the longest wavelength absorption band of the dissolved pyridinium-*N*-phenoxidebetaine dye in a solvent, measured in kcal mol^−1^ [47]. The linear relationship between the maximum emission wavenumbers (ν̃, cm^−1^) and *E*_T_(30) for 2,6-TNS [ν̃ = −256*E*_T_(30) + 36431.5, *R*^2^ = 0.963] and 1.8-ANS [ν̃ = −159.8*E*_T_(30) + 29602.9, *R*^2^ = 0.986] were established, respectively, by plotting the maximum emission wavenumbers of 2,6-TNS/1,8-ANS in a set of solvents as a function of solvent polarity *E*_T_(30) (Figure 1e and 1f). *E*_T_(30) values for the involved solvents (water, methanol, ethanol, propanol, butanol, glycerol and ethylene glycol) and the maximum emission wavenumbers of 2,6-TNS/1,8-ANS were extracted from references [42,47]. The *E*_T_(30) value of GC5A was calculated using the emission maximum of 2,6-TNS/1,8-ANS upon complexation of GC5A, as 51.4 based on 2,6-TNS and as 52.0 based on 1,8-ANS. Both of the calculated results resemble the polarity of ethanol with an *E*_T_(30) value of 51.9. According to maximum emission wavenumbers of 2,6-TNS/1,8-ANS upon complexation with various hosts which have been reported in the literature, *E*_T_(30) values were summarized in Table 2. The polarity of the GC5A cavity is similar to that of the choline-modified calix[5]arene pentadodecyl ether (AmC5A) and is less polar than those of CDs. To be noted, as the fluorescent dyes are immersed to different degrees in cavities, the calculated polarities are also influenced by the position of the dyes inside the macrocyclic hosts [10]. The complexation of GC5A with these naphthalenesulfonic acids generally shows large binding affinities and a prominent fluorescence enhancement. Differing from the complexation-induced quenching of ACQ dyes, the fluorescence enhancement of ICT dyes upon complexation with GC5A would benefit for substrate-selective STA, realizing the desired switch-on sensing (Scheme 1). Besides, they are an eminent matrix for constructing organic luminescent materials and could be engaged in the manufacturing of high-performance supramolecular dye lasers [24,48].

**Table 2:** Binding constants and photophysical properties of host–guest complexes and the calculated polarities of macrocyclic hosts.

Dye	Host	*K*_a_ [M^−1^]	*I*_bound_/*I*_free_	λ_em_ [nm]	*E*_T_(30)	Ref.

1,8-ANS	GC5A	(3.0 ± 0.6) × 10^6^	25	470	52.0	this work
	α-CD	≤20	1.6	500	60.0	[10]
	β-CD	100	2.4	510	62.5	[10]
	AmC5A	3.2 × 10^6^	155	465	50.6	[24]
2,6-TNS	GC5A	(4.4 ± 1.6) × 10^6^	39	430	51.4	this work
	β-CD	3.7 × 10^3^	16	483	61.3	[10]

### The complexation of GC5A with AIE dye

**Figure 2:**
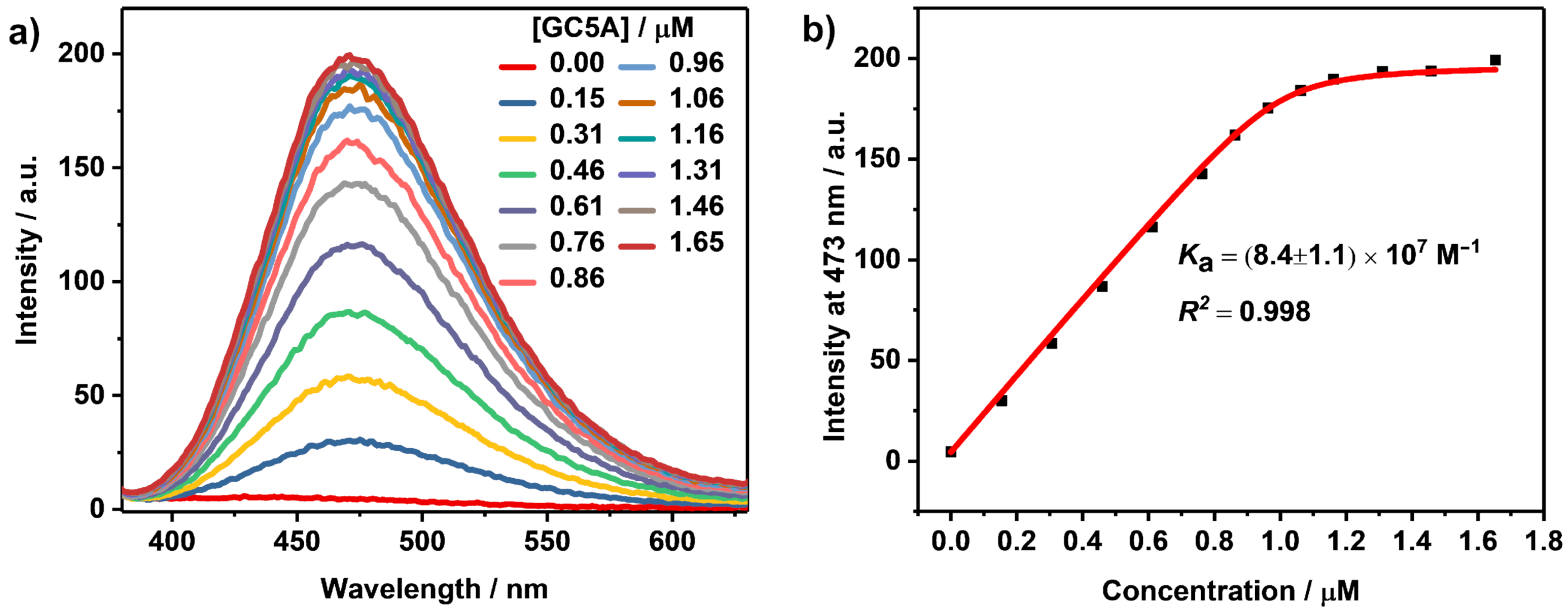
(a) Direct fluorescence titration (λ_ex_ = 327 nm) of P-TPE (1.0 μM) with GC5A in HEPES buffer (10 mM, pH 7.4) at 25 °C and (b) the associated titration curve (λ_em_ = 473 nm) fitting according to a 1:1 binding stoichiometry.

Opposite to ACQ, AIE is a photophysical phenomenon in such way that luminescent fluorophores are non-emissive as isolated molecules but become highly luminescent upon aggregate formation [49]. Once reported by Tang’s group [50], AIE luminogens (AIEgens) have attracted great attention and have been applied in various areas including optoelectronic materials [51–52] and biosensors [49,53]. Tetraphenylethylene (TPE) and its derivatives represent a classical family of AIEgens due to their simple synthesis. In TPE, four phenyl rings are linked to the central olefin through single bonds. As isolated molecule, the phenyl rings have great freedom to rotate or twist against the central olefin stator and thus dissipate the energy of the excited state through non-radiative relaxation pathways which makes the compound non-emissive. However, while forming aggregates, physical constraint is involved by stacking which restricts the intramolecular rotation, therefore leading to a fluorescence enhancement. Herein, the phosphate derivative of TPE, P-TPE, was employed as a model guest to examine the complexation behavior of GC5A with AIEgens. As shown in Figure 2a, a dramatic fluorescence enhancement was observed upon complexation with GC5A. This result is in good accordance with the complexation of *p*-sulfonatocalix[*n*]arenes (SC*n*As, *n* = 4, 5, 6, 8) with ammonium-modified TPE derivatives reported previously by us [54–55]. We assume that the geometrical confinement of P-TPE within GC5A restricts rotational freedom and thus hampers disfavorable non-radiative decay pathways. The inflection point on the titration curve indicates a 1:1 binding stoichiometry between GC5A and P-TPE. *K*_a_ was fitted according to a 1:1 binding model giving a value of (8.4 ± 1.1) × 10^7^ M^−1^ (Figure 2b, Table 1). The emission wavelengths of classical AIEgens are scarcely affected by solvent polarity, which is a typical difference from ICT dyes [56]. The extraordinary fluorescence enhancement of P-TPE upon complexation with GC5A (*I*_bound_/*I*_free_ = 42) would be beneficial for substrate-selective STA, realizing the desired switch-on sensing (Scheme 1). The complexation between calixarenes and AIEgens also offers the opportunity to construct organic luminescent materials and could be engaged in the manufacture of high-performance supramolecular dye lasers [24,48].

### The complexation of GC5A with the two-photon fluorescent probe

**Figure 3:**
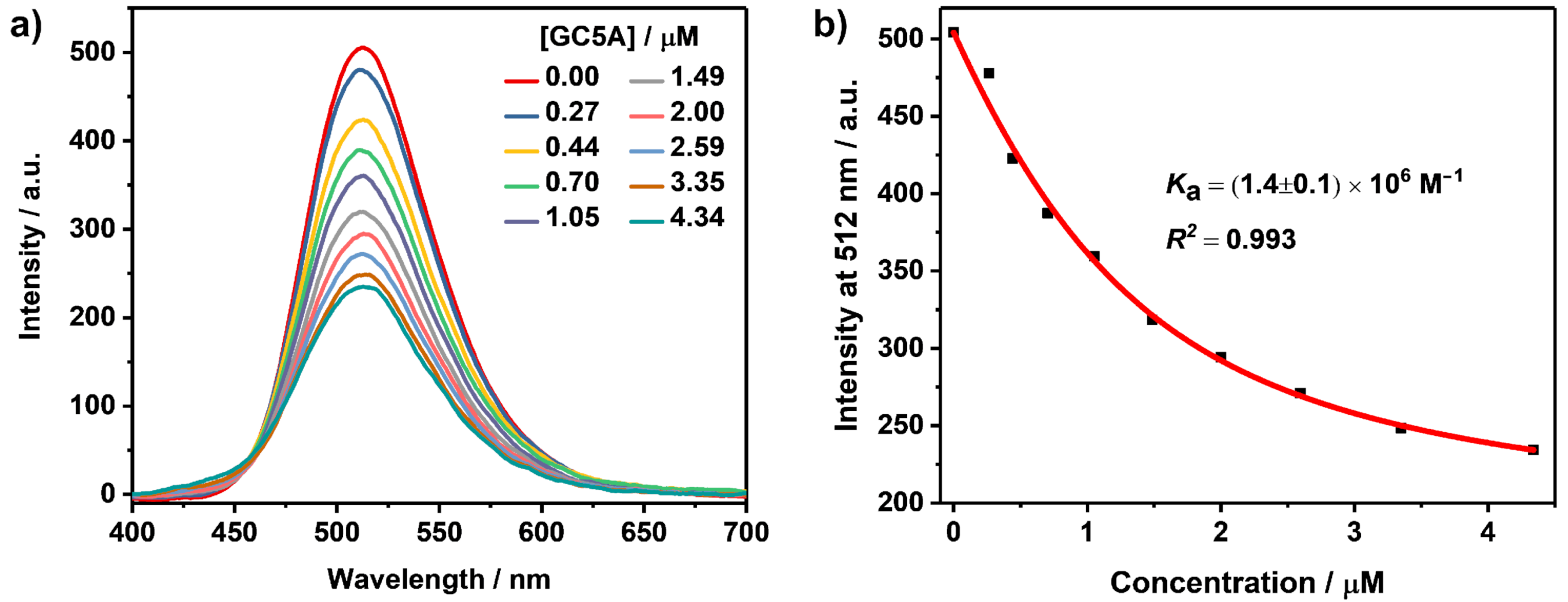
(a) Direct fluorescence titration (λ_ex_ = 371 nm) of TPS (1.0 μM) with GC5A in HEPES buffer (10 mM, pH 7.4) at 25 °C and (b) the associated titration curve (λ_em_ = 512 nm) fitting according to a 1:1 binding stoichiometry.

Two-photon excitation microscopy is a fluorescence imaging technique utilizing near-infrared photons as the excitation source. The fluorescent probe simultaneously absorbs two low energy photons to reach the excited state followed by fluorescence [57]. Acedan (6-acetyl-2-(dimethylamino)naphthalene) is a well-known probe with two-photon absorption that has been utilized in two-photon microscopy imaging. We employed TPS, a derivative of acedan, as a model guest to study the complexation behavior. The gradual addition of GC5A to a TPS solution caused a drastic complexation-induced quenching of fluorescence (Figure 3a). The fluorescence was quenched without a shift of the emission maximum, indicating a tentative PET quenching mechanism, similar to the aforementioned ACQ examples. The data was well fitted by a 1:1 binding model, giving a *K*_a_ value of (1.4 ± 0.1) × 10^6^ M^−1^ (Figure 3b, Table 1). Recently, such host–guest reporter pairs have been applied in cell imaging and even imaging in vivo [16,27,58]. Nau and co-workers reported a SC4A–lucigenin IDA system to monitor the cell-uptake efficiencies of choline, acetylcholine and protamine [58]. Also, we reported a non-covalent fluorescence switch-on strategy for hypoxia imaging in cancer cells by utilizing the complex of a carboxyl-modified azocalix[4]arene with rhodamine 123 as the reporter pair [16]. Consequently, with regard to bioimaging, the study of the complexation between macrocycles and two-photon probes is meaningful as two-photon excitation microscopy exhibits several distinct advantages including a deeper tissue penetration, higher spacial resolution and reduced photodamage of tissue [59].

### The complexation of GC5A with the luminescent transition-metal complex

**Figure 4:**
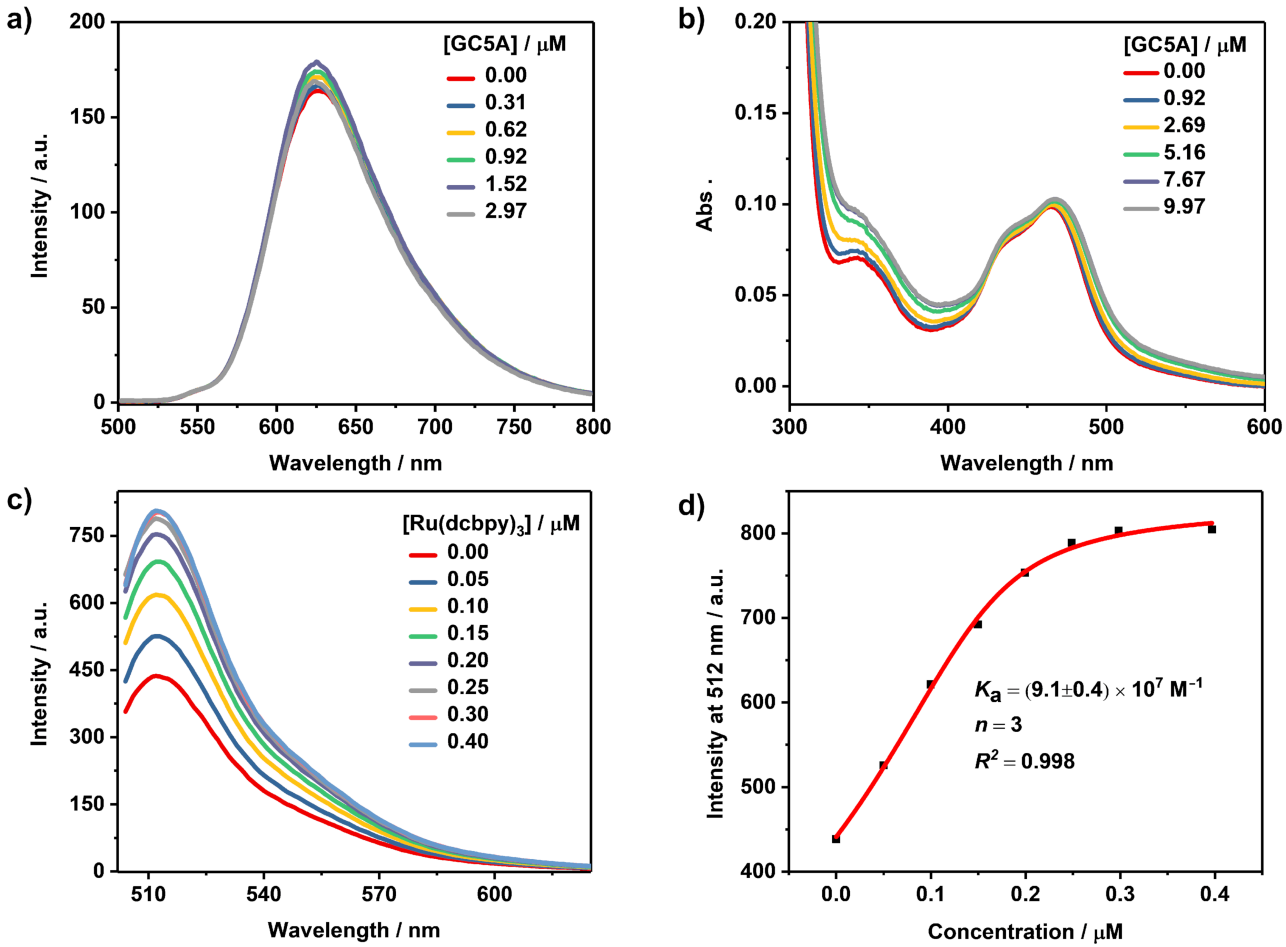
(a) Direct fluorescence titration (λ_ex_ = 465 nm) of Ru(dcbpy)_3_ (1.0 μM) with GC5A. (b) Direct absorption titration of Ru(dcbpy)_3_ (5.0 μM) with GC5A. (c) Competitive fluorescence titration of GC5A–Fl (0.5/0.5 μM) with Ru(dcbpy)_3_, λ_ex_ = 500 nm. (d) The associated titration curve at λ_em_ = 512 nm fitting according to a *n*:1 competitive binding model. All measurements were performed in HEPES buffer (10 mM, pH 7.4) at 25 °C.

Luminescent transition-metal complexes, especially those with ruthenium (Ru), are of great importance owing to their well-documented chemical stability, abundant excited-state photophysics, redox behavior and utilizable luminescence properties [60–61]. These Ru complexes exhibit transitions involving charge transfer from the metal-centered d orbital to the ligand p orbital, commonly known as metal-to-ligand charge transfer (MLCT). Upon excitation, the excited singlet state ^1^MLCT can undergo ultrafast intersystem crossing leading to the formation of the triplet state ^3^MLCT which could emit phosphorescence. Ru(dcbpy)_3_ was employed as a representative candidate in this work to study the photophysical response upon complexation with GC5A. Almost no appreciable luminescence alternation was observed upon gradual addition of GC5A to the Ru(dcbpy)_3_ solution (Figure 4a). The absorption of the MLCT band in the visible region was slightly altered by GC5A (Figure 4b). We therefore employed a competitive fluorescence titration to investigate the host–guest complexation between GC5A and Ru(dcbpy)_3_. The displacement of GC5A–Fl by gradual addition of Ru(dcbpy)_3_ resulted in the regeneration of the intrinsic emission of Fl, implying the formation of the GC5A–Ru(dcbpy)_3_ complex (Figure 4c). The data was well fitted by a *n*:1 competitive binding model, giving a *K*_a_ value of (9.1 ± 0.4) × 10^7^ M^−1^ (Figure 4d, Table 1). The *n* value was fitted as 3, indicating the formation of a 3:1 host–guest complex. We inferred that each 4,4'-dicarboxylic acid-2,2'-bipyridine ligand interacts with one GC5A through salt bridge interaction between a carboxyl anion and guanidinium cation. The photophysical behavior of Ru(II) complexes affected by calixarenes has been studied by different groups. Kirsch-De Mesmaeker and co-workers reported that the luminescence of [Ru(TAP)_2_(phen)]^2+^ (TAP = 1,4,5,8-tetraazaphenanthrene, phen = 1,10-phenanthroline) complex could be quenched by the phenol moieties of a covalent linked calixarene via proton-coupled electron transfer [62]. Kitamura and co-workers reported that the complexation of SC4A could quench the luminescence of tris(2,2'-bipyridine)Ru(II) dichloride (Ru(bpy)_3_), where SC4A serves as a PET quencher [63]. Shinkai and co-workers reported that the inclusion of Ru(bpy)_3_ into the hydrophobic cavity of SC8A led to a considerable luminescence enhancement [64]. That is, the photophysical behavior of Ru(II) complexes is rather complicated upon either covalently linking or non-covalently binding with calixarenes. In our present case, the GC5A–Ru(dcbpy)_3_ complex forms indeed, but the corresponding luminescence remains nearly unaltered. On one hand, Ru(dcbpy)_3_ is too large to be included into the cavity of GC5A, so the luminescence alternation by a hydrophobic effect could be excluded. On the other hand, Ru(dcbpy)_3_ locates at the upper rim of GC5A via salt bridge interactions, so the distance between the host and guest is too long for PET to occur. As well-known, Ru(II) complexes have been widely used in constructing dye-sensitized TiO_2_ solar cells [60]. The strong complexation between GC5A and Ru(dcbpy)_3_ provides an alternative way to non-covalently anchoring TiO_2_ and Ru(dcbpy)_3_, especially with the photophysical property unaltered. Moreover, the interface modification of TiO_2_ by calixarene derivatives can produce an interface energy barrier that could suppress back-electron transportation and/or charge recombination between the photoanode and the photosensitizer and thus improve the photovoltaic conversion efficiency [65].

## Conclusion

In summary, we have investigated the complexation behavior of GC5A with several classical luminescent dyes and analyzed their complexation-induced photophysical changes. GC5A affords strong binding (10^6^–10^8^ M^−1^) to all dyes employed (Table 1), indicating its privileged ability to form stable inclusion complexes with a variety of guest molecules. As for Fl, EY, RB, TPPS, AlPcS_4_ and TPS, a drastic complexation-induced fluorescence quenching without wavelength shifting was observed, which was assumed as the PET mechanism. 2,6-TNS and 1,8-ANS show a pronounced fluorescence enhancement upon complexation and their emission wavelengths experience hypsochromic shifts. On account of the solvatochromic effect, they have been used to estimate the inner cavity polarity of GC5A. The complexation-induced fluorescence enhancement was also observed for P-TPE due to the restriction of intramolecular rotation. GC5A interacts with Ru(dcbpy)_3_ strongly, but does not give rise to appreciable emission alternation.

With these fundamental but important photophysical data in hand, several potential applications can be envisaged. Foremost, in the field of fluorescence sensing, we established a toolbox of reporter pairs. One can always achieve the desired switch-on sensing by screening suitable reporter pairs in any case of IDA, product-selective and substrate-selective STA. A switch-on signal is more favored because quenching effects not relevant to indicator displacement may generate false positives results in “switch-off” sensing [66]. Moreover, these reporter pairs could be used in very dilute solutions benefiting from the high affinities and the corresponding drastic luminescence responses, which are desirable from the viewpoints of economy, sensitivity and interference. When expanding these supramolecular assay strategies to bioimaging in cells and in vivo, two-photon reporter dyes exhibit advantages of deeper tissue penetration, higher spacial resolution and reduced photodamage of tissue. In the field of photodynamic therapy, activatable phototheranostics could be realized via BDA when the complexed dyes are PSs, resulting in the lesion-selective imaging and targeted therapy concurrently. Besides biomedical applications, the complexation of macrocyclic receptors with dyes offers the opportunity in constructing advanced organic luminescent materials, high-performance supramolecular dye lasers, and dye-sensitized solar cells. Although in our present study GC5A has served as a specific test case for molecular recognition of various dyes, such an investigation is inspirable for other artificial receptors, especially those of new analogues [67–72]. We believe that the complexation of artificial receptors with dyes will be still an active research area in the future, and there will be identified abundant applications in diverse disciplines.

## Experimental

### Materials

All reagents and solvents were commercially available and used as received unless otherwise specified. Fluorescein (Fl) was purchased from Sigma-Aldrich, 1-anilinonaphthalene-8-sulfonic acid (1,8-ANS) and 2-(*p*-toluidinyl)naphthalene-6-sulfonic acid (2,6-TNS) were purchased from Tokyo Chemical Industry. Ru(dcbpy)_3_ was purchased from Yuanye Bio-Technology Co., Ltd (Shanghai, China), GC5A [26], P-TPE [73] and TPS [74] were prepared according to the previous literature procedures.

### Samples

The HEPES buffer solution of pH 7.4 was prepared by dissolving 2.38 g of 4-(2-hydroxyethyl)piperazine-1-ethanesulfonic acid (HEPES) in approximate 900 mL double-distilled water. After titration to pH 7.4 at 25 °C with NaOH the volume of the solution was brought to 1000 mL with double-distilled water. The pH value of the buffer solution was then verified on a pH-meter calibrated with three standard buffer solutions. All fluorescence and UV–vis titrations were measured in HEPES buffer (10 mM, pH 7.4) at 25 °C.

### Instruments

Steady-state fluorescence measurements were recorded in a conventional quartz cuvette (light path 10 mm) on a Cary Eclipse equipped with a Cary single-cuvette peltier accessory. UV–vis spectra were recorded in a quartz cuvette (light path 10 mm) on a Shimadzu UV–vis spectrophotometer (UV-2450) equipped with a dual cuvette peltier accessory and a temperature controller (TCC-240A).

### Statistical analysis

All fittings were performed in a nonlinear manner [8]. All mean values from fluorescence titrations were determined from at least three experiments and errors were given as standard deviation.
